# Extraction of Needles, &c., from the Human Body

**Published:** 1856-10

**Authors:** Elisha P. Fearing

**Affiliations:** Nantucket


*Extraction of Needles, &c., from the Human Body.* By ELISHA P. FEARING, M. D., of Nantucket.

[Communicated for the Boston Medical and Surgical Journal.]

In July, 1855, I was called to a single woman, aged 44 years. She was quite fleshy. She complained of great pain and tenderness in the lower part of the abdomen. The lady with whom she had resided for the last fifteen years, informed me that she was insane about ten years ago, and a part of the time in close confinement, but since then was thought to have been rational; that for several years past she had had frequent turns of vomiting a substance about as thick as paint, of a chocolate color (in the opinion of the medical attendant, not blood), and large quantities of bloody pus and other matters; that she had discharged something of the kind *per annum*, and that not long before I was called she had discharged, and had taken, from the rectum *several pounds of a light mahogany-colored substance, in masses or lumps*, of various forms and sizes; also, that about the same time she was troubled with a hard and rather painful swelling in the region of the sigmoid flexure, attended with a sense of weight and dragging down, especially when lying on the opposite side; and that the swelling and pain had gradually subsided as the lumps were discharged.

Believing there were more of these lumps, I prescribed an active cathartic. The first discharges were feculent, followed by more of the same kind of lumps; these last completely filled up the rectum, and caused great suffering. It was with difficulty I removed the largest, which measured *six inches in circumference.* Upon the surface of them was some mucus, and lard, which was used to facilitate their removal. They had evidently been permeated by an oil. Nothing of the kind has since been discharged. A specimen has been analyzed by C. T. Jackson, M. D., State Assayer, and the result at which he arrived is, that "this mass of matter is *dried brown ochre paint.*" He "detected linseed oil."

A few days after the cleaning out of the paint (so called), I removed a common sewing needle, about an inch below the ensiform process. She has since suffered much from nervous irritation, caused by numerous needles, as the event has proved. For, during the last twelve months, *one hundred and twenty-three needles, twelve halves or fractions of needles, and two headless pins, have been extracted.* A large portion of them were taken out from the region of the stomach and abdomen, following in the track of the colon, and many in the immediate neighborhood of the sigmoid flexure. They have also been extracted from other parts of the body and a small number from the limbs; viz., just below the breast, back, loins, just below the left hip, upper and inner part of the thigh, labia pudendi, urethra, perinæum and sphincter ani. One only has been removed from below the knee. One of the largest needles was removed this day, from just below the navel; it has troubled her for a long time, and was situated deeply and obliquely, with the point towards the surface, the point having been bent in the extraction. Some few more can be just felt, but they are too deep to be extracted at present.

The needles varied in size, and were found in different positions; many were perpendicular to the axis of the body, with points presenting—others more or less oblique—some with eyes broken, some with points broken, and a few without either points or eyes. Probably some were broken off when extracted, as were some of the needles. A few of them have undergone little or no change; but by far the largest number are slightly oxidized, having lost their brightness and become brittle; others are more or less corroded. This difference may be owing to the degree of purity of the metal and their locality.

The motion of the needles, no doubt, depended very much upon their situation, and the action of the muscles; for strong muscular action (no matter from what cause) was almost sure to bring forward a crop of needles—that is, force them nearer to the surface, some very near, while others were scarcely perceptible, requiring a rather tedious and painful operation. Eight is the largest number extracted in one day, or at one visit.

A question naturally arises, whether the needles and paint were swallowed, or introduced from without? This question cannot be satisfactorily solved, for I have not been able to obtain information affording the least light in regard to this subject, and must therefore rely upon circumstances and facts as they have become manifest.

Not the least mark or trace of a needle, or any other thing, having been forced through the skin from without, has been discovered, notwithstanding the frequent minute examinations made for that purpose; and within twenty-four hours after such examinations, several needles have been taken out from the part of the body examined, having the same blackened appearance. Had they, a few days previous, been forced through the skin, it would seem almost impossible that the trick should have escaped detection. There are other facts and circumstances which would seem to sustain both sides of this question, but for the want of time and space they must be omitted.

Taking all things into the account, I am inclined to the belief that she did swallow, at least, a portion of the needles (probably in papers), and large quantities of the paint, and did introduce some from without, and, in fact, stuff herself not only with these articles, but with some small pebbles of quartz, which had been taken from the vagina before I saw her, and with whatever else came to hand. Whether sane or not at the time, is not known, in all probability not; for of all things needles would be about the last any sane person would think of swallowing, or forcing in from without in such numbers. It has occurred to me, whether, if the needles were swallowed, they wight not have remained a long time in the patient, and thus been preserved, or protected from the corrosive effect of surrounding substances. I have recently seen a part of a needle, which was taken from the forefinger, nearly six months after it was accidentally forced in. It had not changed at all, except having lost its brightness. I have scarcely a doubt that they may, under some circumstances, remain in the body for a long time, perhaps for years, without much change by oxidation. In regard to this very extraordinary case of needles, I will, in conclusion, just observe that, no doubt, a very small number of needles remain to be extracted, which are not accessible at present.

The needles and a specimen of the paint have been deposited in the Cabinet of the Boston Society for Medical Improvement.

*Nantucket, Mass., July 26th,* 1856.

